# Expression of concern: measles-mumps-rubella vaccination timing and autism among young African American boys: a reanalysis of CDC data

**DOI:** 10.1186/2047-9158-3-18

**Published:** 2014-08-29

**Affiliations:** 1 Floor 6, 236 Gray's Inn Road, London, WC1X 8HB, UK

## Abstract

The Publisher of this article [1] has serious concerns about the validity of its conclusions because of possible undeclared competing interests of the author and peer reviewers. The matter is undergoing investigation. In the meantime, readers are advised to treat the reported conclusions of this study with caution.

Further action will be taken, if appropriate, once our investigation is complete.

## Comment on

Brian Hooker. Measles-mumps-rubella vaccination timing and autism among young African American boys: a reanalysis of CDC data. *Translational Neurodegeneration* 2014, **3**:16.

## References

[B1] Hooker BS (2014). Measles-mumps-rubella vaccination timing and autism among young
African American boys: a reanalysis of CDC data. Translational Neurodegeneration.

